# Electromyography analysis of rotator cuff activation while driving

**DOI:** 10.1016/j.jseint.2025.101411

**Published:** 2025-12-01

**Authors:** Megan C. Paulus, Brent B. Wiesel, Lauren W. Bierman, Tom C. Galetti, Shaan S. Nagda, Kevin F. Fitzpatrick, Sameer H. Nagda

**Affiliations:** a Stony Brook Orthopaedic Associates, Stony Brook, NY, USA; b Medstar Georgetown University Hospital, Washington, DC, USA; c Associated Eye Surgeons, Plymouth, MA, USA; d University of California, Berkeley, CA, USA; e West Potomac High School, Alexandria, VA, USA; f Mount Vernon Rehabilitation Medicine Associates, Inova Rehabilitation Center, Alexandria, VA, USA; g Anderson Orthopaedic Clinic, Alexandria, VA, USA

**Keywords:** Rotator cuff repair, Rotator cuff tear, Muscle activation, Driving guidelines, EMG, Shoulder arthroscopy

## Abstract

**Background:**

A common concern of patients after rotator cuff repair is when they can drive. The purpose of this study is to evaluate the activation of the rotator cuff while driving to help guide surgeon recommendations.

**Methods:**

A computerized driving simulator was used by 16 volunteers who performed a series of turns with their hands in different positions on the steering wheel. Muscle activity of the supraspinatus, infraspinatus, and biceps was recorded using electromyography. Muscle activity was also recorded while closing the door, fastening the seat belt, and turning a key in the ignition and quantified by a board-certified electromyographer.

**Results:**

For the right supraspinatus, infraspinatus, and biceps muscles, there was statistically significant higher level of activation when the right hand was in the 12 o'clock position and the 3 o'clock position as compared with the 6 o'clock positions. This was the same for the left side with the left hand in the 12 o'clock and 9 o'clock positions compared to the 6 o'clock positions (*P* < .003). Activity of all muscles was minimal when the car door was closed with the opposite hand. Fastening the seat belt and turning the key in the ignition also demonstrated rotator cuff activation.

**Conclusion:**

Rotator cuff activity during driving can be minimized by closing the door using the nonoperative arm and driving with the nonoperative arm in the 12 o'clock position with the operative arm at the side and holding the wheel at the 6 o'clock position.

Each year in the United States, approximately 200,000 patients undergo rotator cuff repair (RCR), and an additional 400,000 patients have surgery for rotator cuff tendonitis or for partial tears. The national rates of RCR have increased dramatically over the last decade.^4^ Postoperatively, patients are immobilized in a sling for up to 6 weeks to allow for healing to occur.^12^ During this period, patients commonly ask when they are able to return to their activities of daily living, including driving, and driving is 1 of the 5 most difficult activities for shoulder surgery patients to do while immobilized.^6^ Despite driving's importance and difficulty to patients, no clearly established guidelines exist to help determine when a patient can return to driving while neither endangering their repair nor posing a risk to other drivers.^13^

Patient reported data on return to driving after shoulder surgery reflects the ambiguity on the subject. One review reports average return to driving at two months after RCR and 1-3 months after total shoulder arthroplasty.^5^ However, another study showed a broader range for RCR patients to return to driving — from the day of surgery up to four months postoperatively.^7^ Furthermore, studies have shown that reported return to driving did not correspond with perceived safety or preparedness.^1^^,^^7^ An evaluation of simulated driving performance after total shoulder arthroplasty demonstrated a return to preoperative driving levels at six weeks, while arthroscopic shoulder surgery patients showed an increased number of collisions driving at six weeks postoperatively when compared with preoperative levels.^10^^,^^11^

To date, no study has evaluated the function and activation of the rotator cuff during driving with the use of electromyography (EMG). As return to driving is multifactorial and protection of the repair is 1 such factor, knowledge of rotator cuff activation may help guide the surgeon's recommendations regarding when a patient should drive after RCR. The purpose of this study is to evaluate the activation of the supraspinatus, infraspinatus, and biceps muscles while driving. Understanding how to best protect the RCR is 1 factor that can help guide the surgeon's recommendation as to when the patient may begin driving. We hypothesize that the rotator cuff and biceps will be active during routine driving, and this activation will vary based on the position of the hands on the steering wheel.

## Materials and methods

IRB approval was obtained, and informed consent was obtained from participants. A computerized driving simulator (Drivesquare, Alexandria, VA, USA) was used in a standard 4-door sedan. This simulator places a rotating platform under both front wheels of a standard car allowing the subject to turn the steering wheel as if driving and the simulator to recreate normal driving situations. Sixteen healthy volunteers (8 males and 8 females) aged 24 to 30 with no history of shoulder pathology performed a series of driving maneuvers while muscle activity of the supraspinatus, infraspinatus, and biceps was recorded and interpreted by a board-certified electromyographer using EMG to quantify motor unit activity.

### Use of EMG

In order to minimize the chances of recording signals from nearby muscles, needle EMG was used for the supraspinatus and infraspinatus muscles, while surface EMG was used to record biceps activity. Needle EMG was utilized for the supraspinatus and infraspinatus muscles, as surface EMG may have made it impossible to distinguish their activity from that of the trapezius muscles (which overlay both the supraspinatus and infraspinatus). Thus, for the supraspinatus and infraspinatus muscles, a standard monopolar 1.5-inch, 28-gauge EMG needle electrode (Ambu, Ballerup, Denmark) was inserted into the respective muscle and connected to a standard EMG machine (Natus, Pleasanton, CA, USA). For the biceps brachii, a standard disc electrode (Natus, Pleasanton, CA, USA) was attached to the patient's skin over the belly of the muscle and attached to the same EMG machine. The biceps brachii is a relatively superficial muscle with a fairly low risk of recording from nearby muscles. For these reasons, surface EMG was considered appropriate for the biceps muscle.

Prior to initiation of driving simulation, proper electrode placement was confirmed by asking the subject to abduct the shoulder (supraspinatus), externally rotate the shoulder (infraspinatus), or flex the elbow (biceps) with the electrode in place. For each muscle, a baseline for maximal motor unit activity (full interference pattern) was established by asking the subject to provide maximal contraction against resistance. During the recording phase, when necessary, the EMG needle electrode was repositioned by the electromyographer between movements to ensure electrode placement was maintained in the muscle being recorded. Muscle activity was also recorded for predriving activities: closing the door, fastening the seat belt, and turning a key in the ignition. During the driving portion, muscle activity was recorded while making a series of right and left turns. First, the hands were placed in the 9 and 3 o'clock positions as recommended in most states. Next, the left hand was placed in the 12 o'clock position with the right hand in the 6 o'clock position. Then the right hand was placed in the 12 o'clock position and the left hand in the 6 o'clock position. When the hands were in the 9 and 3 o'clock positions, both hands worked in concert with one another for the different driving maneuvers. For the 12 and 6 o'clock positions, the hand in the 12 o'clock position was most involved in turning the wheel while the hand in the 6 o'clock position was used to assist in the turn and help control the wheel. These positions were selected based on years of patient feedback of how driving was successfully performed with injured shoulders. Each driver was instructed to operate the wheel in these positions. Each turn was repeated 5 times with each hand position, and the average EMG activity was calculated. All maneuvers and positions were performed separately for each muscle tested; therefore, for each patient, the driving protocol was performed 6 times.

### Grading scale

In order to quantify the muscle activity recorded by EMG, the study authors devised and used a grading scale for the purposes of this study. Each repetition of each driving maneuver was graded from 0 to 4 for each muscle studied. A grade of 0 represents no motor unit activity. A grade of 1 represents 1, 2, or 3 distinct motor units firing simultaneously. A grade of 2 represents more than 3 simultaneous motor units, with clear baseline voltage recorded between motor units. Grade 3 represents less than full interference pattern, but no baseline voltage recorded between motor units. Grade 4 represents a full interference pattern.

### Statistical analysis

Statistical comparisons were made using the one-sample Wilcoxon-Mann-Whitney test applied to within-subject differences in EMG activity. Differences were taken between turns made with the hand at 6 o'clock and the same turn made with the hand at a higher position (eg, turning left with the right hand at 6′ o'clock vs. the same turn with right hand at 12 o'clock). These comparisons were chosen a priori, based on the main hypothesis. This leads to 4 comparisons for each muscle (eg, right biceps activity was compared with the right hand at 6′ o'clock vs. 3 o'clock and 12 o'clock, on left and right turns), for 6 muscles (biceps, infraspinatus, and supraspinatus for both right and left arms). Because 24 comparisons were made, a *P* value of 0.002135 applied to each test will control for multiple comparisons.

## Results

For the right supraspinatus, infraspinatus, and biceps muscles, EMG analysis demonstrated a significantly higher level of activation when the right hand was in the 12 and 3 o'clock positions as compared with the 6 o'clock position. Both right and left turns resulted in similar patterns of muscle activation. Similar results were seen on the left side, where significantly higher levels of EMG activity were seen in all 3 muscles with the left hand in the 12 o'clock and 9 o'clock positions compared to the 6 o'clock position (Table I). In all cases, this difference was statistically significant (*P* < .00213).

**Table I:** Muscle activation during driving.

Muscle	Hand positions					
	9 o'clock & 3 o'clock		12 o'clock (left) & 6 o'clock (right)		12 o'clock (right) & 6 o'clock (left)	
	Right turn	Left turn	Right turn	Left turn	Right turn	Left turn
R supraspinatus	2.79	2.77	0.76∗	1.00∗	2.95	2.99
R infraspinatus	2.73	2.92	0.65∗	0.70∗	3.13	3.10
R biceps	2.46	2.81	1.15∗	1.24∗	3.23	3.18
L supraspinatus	2.92	2.75	3.15	3.00	0.90∗	0.64∗
L infraspinatus	2.92	2.96	3.27	3.14	0.88∗	0.97∗
L biceps	2.94	2.80	2.91	3.01	1.10∗	1.19∗

*L*, left; *R*, right.

∗ Statistical significance noted for all comparisons (*P* < .003).

Activity of all muscles was minimal when the car door was closed with the opposite hand. Fastening the seat belt resulted in high activity in all 3 muscles bilaterally. Turning the key in the ignition to start the car mildly activated the right supraspinatus and infraspinatus with slightly higher activity in the biceps muscle (Table II).

**Table II:** Muscle activation in activities to prepare for driving.

Muscle	Activities involved in preparation for driving			
	Close door with left arm	Close door with right arm	Turn key in ignition	Fasten seatbelt
R supraspinatus	1.38	-	2.31	3.00
R infraspinatus	0.75	-	2.44	2.81
R biceps	0.81	-	2.63	2.91
L supraspinatus	2.63	1.15∗	-	2.56
L infraspinatus	2.75	1.15∗	-	2.88
L biceps	2.63	1.08∗	-	2.88

*L*, left; *R*, right.

∗ Statistical significance noted (*P* < .003).

## Discussion

The decision to allow a patient to drive after RCR is two-fold. First, it is important that the patient is able to drive safely, ensuring they are unimpaired with adequate reaction times. Second, it is necessary that the process of driving does not compromise the repair.

Simulators have been used to assess the effect of upper extremity immobilization on driving, addressing the concern over safety. Two studies have shown that dominant arm immobilization through either a below-elbow cast or a sling has led to less cautious driving, worsened response to hazards, and an increase in the number of collisions.^8^^,^^9^ Similar results with a trend toward decreased driving performance, increased perceived difficulty, and decreased perceived safety with upper extremity immobilization were found in a third study.^3^ Hasan et al studied the driving reaction time in patients before and after arthroscopic shoulder surgery and patients before and after shoulder arthroplasty. They concluded that the patient's ability to avoid collisions was decreased at six weeks after arthroscopy and returned to baseline by 12 weeks.^11^ In the shoulder arthroplasty patients, they found an improvement in driving performance by 12 weeks postoperatively as compared to before surgery.^10^

While the discussed studies evaluated the ability of the patient to drive safely, they did not evaluate the impact of driving on the rotator cuff and therefore driving's potential impact on the healing process. One musculoskeletal modeling study used optical motion tracking markers in subjects driving under varied circumstances. The study found that muscle activation and force were predicted in each of the driving positions within each of the upper extremity muscle groups, and the group warns against repeated high activation and fatigue.^14^ They concluded that most driving conditions cause moderate to high activation of the supraspinatus and deltoid with some moderate activation of the infraspinatus, but they suggested further validation with the use of EMG.

Our study extended this work by using EMG to evaluate the activity of the rotator cuff muscles while driving. We also evaluated the effect of different hand positions on rotator cuff function. We surmised that placing the hand in different positions would have an impact on rotator cuff activation. Our study showed high levels of activity in the supraspinatus, infraspinatus, and biceps with the hands in the 12 o'clock, 9 o'clock, and 3 o'clock positions on the steering wheel. This activity was minimized on each side by placing the hand at the 6 o'clock position on the steering wheel. Our findings are supported in principle by another study that did not use EMG. This study found that patients preferred to drive with the hand ipsilateral to the repaired rotator cuff on the lower portion of the wheel; they noted that right-sided RCR patients increased right hand placement on the upper portion of the steering wheel at week 12 postoperatively, and left-sided RCR patients significantly decreased left-handed upper wheel positioning at week 2.^2^ These results may be explained, in part, by the EMG findings of our study. The information learned from our study in combination with prior studies provides substantial evidence for physicians to consider when evaluating when a patient should drive after rotator cuff surgery.

Our study also demonstrated increased muscle activity while closing the car door, putting the seatbelt on, and turning the key in the ignition. Using the nonoperative arm to close the door will decrease the amount of muscle activity in the operative arm. We did not study activation of the right-sided muscles while trying to close the door with the right arm, as we assumed that patients who underwent surgery on their right arm would not try to close the door with their right arm while sitting in the driver's seat. Turning the key with the right hand may be unavoidable; caution should be taken by patients who underwent right-sided RCR as muscle activity is present during this activity. Push-button starting cars may allow a patient to reach over with the left hand to start the car, but this feature was not included in our study. While we instructed patients to use only the nontested arm to reach for and fasten the belt, relatively high muscle activity was noted on the tested side. This concern for rotator cuff activation would exist even if the patient were a passenger. We were unable to find a way to minimize cuff activation during this activity.

One of the main limitations of this study is quantifying the EMG activity in relation to acceptable muscle activation levels for healing to occur after RCR. While it remains unknown how much activity each different repair will tolerate, we can confidently make the assumption that limiting the EMG activity will provide the least amount of stress on the repaired tissues. Knowledge of muscle activity from this study can help guide surgeons as to when they might feel that a patient can drive without compromising the integrity of the repair. A second limitation is that our study population was much younger than patients who typically undergo rotator cuff surgery. It is possible that older patients may have different muscle recruitment patterns during driving than our younger volunteers. We elected to use younger participants in order to minimize the chance of rotator cuff pathology altering the results. We also used a standard 4-door midsize car. Different results might be noted in a truck or compact car.

In addition, this study did not examine muscle activation neither during sudden sharp turns nor during sudden braking, both of which may occur during the course of driving a car. Last, we did not assess the reaction time or safety of the driver with the hands in different positions. It is unknown whether driving with the hands in the 12 and 6 o'clock positions is as safe as driving in the traditional 9 and 3 o'clock positions. Further research should focus on evaluating driving performance in the postoperative patient with the hands on the wheel in the modified positions suggested from our study. While there are no laws governing hand positioning on the steering wheel, it would be ideal to have the hands in a position that will protect the repair, is comfortable, and allows the patient to drive safely; this study only addressed the first of these concerns. Several of the subjects did comment that they routinely drove with 1 hand at the 12 o'clock position. Anecdotally, many of the senior authors' prior RCR patients have expressed that they elected to drive with their unaffected and affected arms at the 12 and 6 o'clock positions, respectively, to minimize pain while driving in the injured state. We also did not test patients with a sling on, as this is considered driving impaired in most states and is not recommended. Our goal was to establish a predictable pattern of rotator cuff and biceps activation during routine driving. We were unable to assess subscapularis activity, as precise measurement of the subscapularis required needle EMG. We attempted this 1 time; however, it severely altered the subject's ability to manipulate the steering wheel and the electromyographer was unable to keep the needle in place to obtain usable data.

Despite these limitations, our study is the first to document muscle activity patterns of the rotator cuff and biceps while driving. In addition, we revealed optional hand positions on the steering wheel to minimize rotator cuff activation. However, some patients may not be comfortable driving with their hands at 12 o'clock and 6 o'clock, and this may not be the universally safest hand position for driving. Furthermore, it should be stressed that the decision as to when a patient should be allowed to drive is still multifactorial, and the surgeon and patient should consider each unique repair and healing potential as well as pain, stiffness, the distance needed to drive, and traffic patterns, as all of these factors could possibly influence and affect a safe return to driving. Nevertheless, our study provides clinicians with useful information that should be considered when evaluating when a patient may return to driving while maintaining the integrity of the repair.

## Conclusion

This study was conducted to determine rotator cuff muscle activation while driving in order to determine the driving practices that best maintain the integrity of the repair. The study found that the rotator cuff, particularly the supraspinatus and infraspinatus muscles, is extremely active during routine driving. The following recommendations can minimize activation of the cuff during most driving activities: (1) close the door using the nonoperative arm and (2) drive with the nonoperative arm in the 12 o'clock position with the operative arm and at the side while holding the wheel at the 6 o'clock position. While this study can provide insight into a return to driving that maintains the safety of the repair, the decision of when to allow driving after rotator cuff surgery is multifactorial. Further research should attempt to address driver safety and performance after rotator cuff surgery given the information learned and suggested hand placement from our current study.

## Disclaimers:

Funding: This study was funded by a grant supported by Inova Health Systems. The grant money of $8,400 was used to supply a computerized driving simulator (Drivesquare, Alexandria, VA). All authors certify that neither themselves nor any of their affiliations received financial payments or other benefits from any commercial entity related to the subject of this article.

Conflicts of interest: Dr. Wiesel reports publishing royalties, financial or material support from Wolters Kluwer Health - Lippincott Williams & Wilkins outside of the submitted work. Dr. Nagda reports IP Royalties from Aevumed as well as being a paid presenter or speaker and research support from Stryker outside of the submitted work. All the other authors, their immediate families, and any research foundation with which they are affiliated have not received any financial payments or other benefits from any commercial entity related to the subject of this article.
